# Autophagy Alteration in ApoA-I Related Systemic Amyloidosis

**DOI:** 10.3390/ijms23073498

**Published:** 2022-03-23

**Authors:** Rita Del Giudice, Paola Imbimbo, Federico Pietrocola, Isabelle Martins, Fatima Domenica Elisa De Palma, José Manuel Bravo-San Pedro, Guido Kroemer, Maria Chiara Maiuri, Daria Maria Monti

**Affiliations:** 1 Department of Chemical Sciences, University of Napoli Federico II, Complesso Universitario Monte Sant’Angelo, 80126 Napoli, Italy; rita.del-giudice@mau.se (R.D.G.); paola.imbimbo@unina.it (P.I.); 2 Department of Biosciences and Nutrition, Karolinska Institute, 14157 Huddinge, Sweden; federico.pietrocola@gmail.com; 3 Centre de Recherche des Cordeliers, INSERM U1138, Université Paris Cité, Sorbonne Université, 75006 Paris, France; isabelle.martins@inserm.fr (I.M.); depalma@ceinge.unina.it (F.D.E.D.P.); kroemer@orange.fr (G.K.); chiara.maiuri@upmc.fr (M.C.M.); 4 Metabolomics and Cell Biology Platforms, Gustave Roussy Cancer Campus, 94805 Villejuif, France; 5 CEINGE-Biotecnologie Avanzate s.c.a.r.l., 80145 Napoli, Italy; 6 Department of Molecular Medicine and Medical Biotechnologies, University of Napoli Federico II, 80131 Napoli, Italy; 7 Departamento de Fisiologia, Universidad Complutense de Madrid, 28040 Madrid, Spain; 8 Institut Universitaire de France, 75005 Paris, France; 9 Pôle de Biologie, Hôpital Européen Georges Pompidou, Ap-hp, 75015 Paris, France; 10 Pharmacy Department, University of Napoli Federico II, 80131 Napoli, Italy; 11 Istituto Nazionale di Biostrutture e Biosistemi (INBB), 00136 Rome, Italy

**Keywords:** amyloidosis, autophagy, apoptosis, apolipoprotein A-I

## Abstract

Amyloidoses are characterized by the accumulation and aggregation of misfolded proteins into fibrils in different organs, leading to cell death and consequent organ dysfunction. The specific substitution of Leu 75 for Pro in Apolipoprotein A-I protein sequence (ApoA-I; L75P-ApoA-I) results in late onset amyloidosis, where deposition of extracellular protein aggregates damages the normal functions of the liver. In this work, we describe that the autophagic process is inhibited in the presence of the L75P-ApoA-I amyloidogenic variant in stably transfected human hepatocyte carcinoma cells. The L75P-ApoA-I amyloidogenic variant alters the redox status of the cells, resulting into excessive mitochondrial stress and consequent cell death. Moreover, L75P-ApoA-I induces an impairment of the autophagic flux. Pharmacological induction of autophagy or transfection-enforced overexpression of the pro-autophagic transcription factor EB (TFEB) restores proficient proteostasis and reduces oxidative stress in these experimental settings, suggesting that pharmacological stimulation of autophagy could be a promising target to alleviate ApoA-I amyloidosis.

## 1. Introduction

Amyloidosis is a group of heterogeneous diseases caused by protein misfolding, which manifest as neurodegenerative disorders (affecting the central nervous system), or systemic pathologies affecting other vital organs [1,2]. This class of pathologies is characterized by the aggregation of specific misfolded proteins into insoluble fibrils, followed by their aberrant deposition in tissues and organs, which disrupts tissue architecture while compromising physiological functions [3]. Among the human amyloidosis identified so far, a hereditary systemic amyloidosis is caused by mutations in the gene encoding Apolipoprotein A1 (ApoA-I), the major protein in high-density lipoproteins. To date, 23 ApoA-I amyloidogenic mutations have been identified [4,5,6,7,8,9]. These ApoA-I variants are responsible for late onset, autosomal dominant, hereditary systemic amyloidosis, characterized by protein aggregates deposition in heart, liver, kidneys, nerves, ovaries or testes, leading to organ damage and eventually failure ([10] and references therein). Most of the mutations occur as a single-nucleotide substitution and are localized in two main hot spots, encompassing residues 50–93 and 170–178. Intriguingly, although ApoA-I-related amyloidosis is a systemic disease, the localization of the amino acid substitution to either of these two regions dictate which organ/tissue will be predominantly affected. For instance, patients with alterations in the N-terminal region (residues 1–75) mostly show hepatic, renal, and testis involvement, whereas carriers of mutations in residues 173 to 178 mainly develop cardiac, laryngeal and cutaneous amyloidosis [6,11,12,13]. ApoA-I-related amyloidosis represents an intriguing field of study because, despite significant advances in the study of the structure and aggregation propensity of ApoA-I amyloidogenic variants [11,14,15,16,17], (i) the mechanism responsible for the onset and progression of this disease is largely unknown, and (ii) limited therapeutic options are available to counteract the detrimental effect of protein aggregates deposition. Accordingly, a better understanding of the molecular alterations induced by ApoA-I amyloidogenic variants in target cells would be instrumental for the implementation of novel therapeutic approaches aiming to alleviate amyloidosis. 

To this end, we focused on the ApoA-I amyloidogenic variant L75P-ApoA-I, which accounts for the preferential deposition of amyloid fibrils in the liver. Although the worldwide incidence of L75P-ApoA-I related amyloidosis is low, this mutation is highly prevalent in a narrow region of Northern Italy, close to Brescia, where 50 families with carriers have been isolated [18]. The cDNA encoding this fibrillogenic variant has been previously utilized to stably transfect human liver cells. As hepatocytes express endogenous WT-ApoA-I, cells transfected with L75P-ApoA-I simultaneously express both the native protein and the L75P variant, presumably mimicking the heterozygous genotype of human patients [19].

Macroautophagy (henceforth referred to as “autophagy”) is a cellular mechanism responsible for the maintenance of cellular homeostasis, as it is essential for the clearance of damaged organelles and potentially toxic protein aggregates [20,21]. Inefficient autophagy is directly involved in a number of pathologies including hereditary myopathies, chronic inflammation, cancer and neurodegenerative diseases, such as Parkinson’s and Alzheimer’s disease [22,23]. Moreover, the involvement of ApoA-I in regulating autophagy in hepatic steatosis has been recently demonstrated [24,25]. Our results show that the presence of an ApoA-I amyloidogenic variant impairs redox homeostasis and blocks autophagic flux in stably transfected HepG2 cells.

## 2. Results

### 2.1. L75P-ApoA-I Variant Compromises Cellular Redox Homeostasis and Causes Mitochondrial Dysfunction

**Figure 1:**
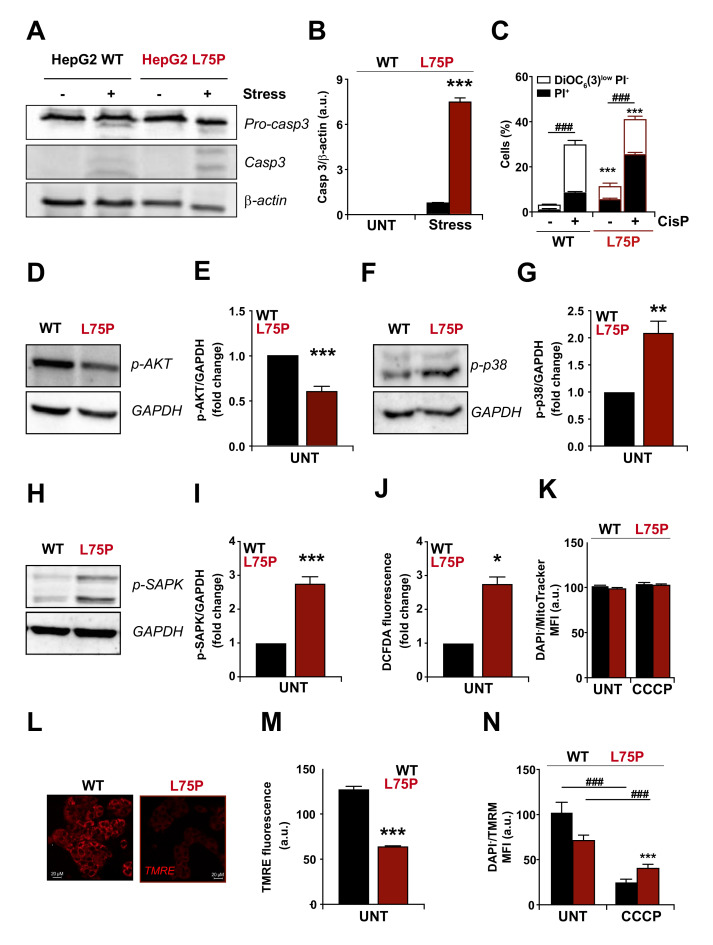
Alteration of cellular redox homeostasis in L75P-ApoA-I expressing cells. (**A**,**B**) Stress-induced apoptosis in L75P-ApoA-I expressing cells. Wild type (WT) or L75P-ApoA-I (L75P) HepG2 cells were cultured in control conditions (UNT) or maintained in FBS-free medium (stress) and analysed by immunoblotting with antibodies specific for caspase-3 (Casp3) and β-actin (loading control). Apoptosis activation coincides with a reduction in the intensity of the pro-caspase 3 band paralleled by an increase of the bands corresponding to the active fragments (the relative densitometry is shown; means ± SD of at least three independent experiments). (**C**) Cytometric quantification of cell death induced by cisplatin treatment (CisP). WT and L75P HepG2 cells were treated, collected, and double stained with PI and DiOC_6_(3) for the detection of dying (DiOC_6_(3)^low^-PI^−^) and dead (PI^+^) cells. (**D**–**I**) Mechanistic insights of L75P-ApoA-I expressing cells. WT and L75P HepG2 cells were cultured in control conditions, followed by the assessment of AKT (**D**,**E**), p38 (**F**,**G**) and SAPK/JNK (**H**,**I**) phosphorylation (the relative densitometry is shown). GAPDH levels were monitored to ensure equal loading of lanes. (**J**–**N**) Expression of L75P-ApoA-I causes significant mitochondrial dysfunction. Intracellular oxidative stress was determinate as presence of cytosolic reactive oxygen species (ROS) and assessed by dichlorodihydrofluorescein (DCFDA) fluorescence intensity in WT and L75P HepG2 cells (**J**). Cells were cultured for 1 h with carbonyl cyanide 3-chlorophenylhydrazone (CCCP, 10 µM) then subjected to staining with MitoTracker and 4′,6-diamidino-2-fenilindol (DAPI) (**K**). ROS measurements are from three independent experiments made in duplicates (means ± SEM). Changes in mitochondrial membrane potential (Δψm) were measured in wild type and L75P-ApoA-I HepG2 cells using tetramethylrhodamine ethyl ester (TMRM) TMRE probe (**L**,**M**). Since TMRE accumulates only in mitochondria with high Δψm, the change in TMRE fluorescence intensity indicates a fall in Δψm as a result of ApoA-I expression. Images are from one experiment and representative of three independent experiments made in triplicates. Cells were cultured for 1 h CCCP (10 µM) then subjected to staining with TMRM and DAPI (**N**). If not specified, the panels in this figure are shown as means ± SD of at least three independent experiments. Asterisks symbols indicate significant changes with respect to WT cells (* *p* < 0.05, ** *p* < 0.01, *** *p* < 0.001). Hash symbols indicate significant changes respect UNT condition (### *p* < 0.001). a.u. arbitrary units; MFI: Mean Fluorescence Intensity.

We previously demonstrated that L75P-ApoA-I transfectants are more sensitive than control cells to serum withdrawal and hypothesized that cell death occurred via apoptosis [19]. To confirm this hypothesis, we analysed signs of apoptosis in HepG2 cells expressing the amyloidogenic variant L75P-ApoA-I. L75P-ApoA-I HepG2 cells, as well as in their wild type counterpart, were exposed to different types of stress. Cells were incubated in the absence of serum for 48 h or treated with cisplatin, and then cell lysates were analysed by immunoblotting using an anti-cleaved caspase 3 antibody and by flow cytometry to double stained with the vital dye propidium iodine (PI) and the mitochondrial transmembrane (Δψm) probe DiOC_6_(3). As shown in Figure 1A–C, we found that cells overexpressing the amyloidogenic form L75P-ApoA-I were characterized by enhanced apoptosis levels, both at basal levels and after death stimuli (serum withdrawn), compared to wild type cells measured by the active form of pro-caspase 3 (Figure 1A,B) and cytometric detection of dying (DiOC_6_(3)^low^ PI^−^) and dead (PI^+^) cells (Figure 1C).

We previously reported that, in L75P-ApoA-I transfectants, the amyloidogenic variant is retained within the cell, representing a stress factor [19]. Thus, we indirectly analysed the redox status of L75P-ApoA-I expressing cells by immunoblotting analysis of serine/threonine kinase protein kinase B (best known as AKT), MAP kinase p38 (MAPK) and the stress-activated protein kinase/Jun-amino-terminal kinase (SAPK/JNK) (Figure 1D–I). AKT normally promotes cell survival by inhibiting apoptosis through phosphorylation and inactivation of several targets and is activated by insulin and various growth and survival factors [26]. p38 is activated by a variety of cellular stresses, UV light and growth factors, whereas SAPK/JNK is potently and preferentially activated by different environmental stresses and, in some instances, growth factors [27,28,29]. In L75P-ApoA-I transfected cells, AKT phosphorylation level was significantly decreased (about 50%) compared to control wild type cells (Figure 1D,E), whereas the phosphorylation levels of both p38 (Figure 1F,G) and SAPK/JNK (Figure 1H,I) were significantly higher (2.2- and 2.9-fold increase, respectively) compared to control cells, thus suggesting that the presence of the L75P amyloidogenic variant into the cell might cause an imbalance in the redox status.

In order to confirm that in cells expressing the ApoA-I amyloidogenic variant the redox homeostasis is altered, we evaluated the production of intracellular reactive oxygen species (ROS) by incubating cells in the presence of dichlorodihydrofluorescein (H_2_-DCFDA, which is non-fluorescent) and then measuring its ROS-dependent oxidation to DCFDA, which is fluorescent. ROS production was 1.6-times higher in L75P-ApoA-I stably transfected HepG2 cells than in wild type HepG2 cells (Figure 1J). Because the abnormal accumulation of cytosolic proteins elicits a direct stress to the cells [30] and oxidative stress is often linked to mitochondrial dysfunctions [31,32], we evaluated the number of mitochondria by using Mitotracker labelling and the mitochondrial membrane potential using tetramethylrhodamine ethyl ester (TMRE) dye in both cell lines (Figure 1K–N). We found that the number of mitochondria is not impacted by the L75P-ApoA-I mutation (Figure 1K). However, the presence of the L75P amyloidogenic variant induced a strong depolarization of mitochondria in HepG2 cells, as demonstrated by the 50% decrease in the TMRE signal (Figure 1L,M). L75P-ApoA-I stably transfected HepG2 cells displayed lower levels of TMRM compared to HepG2 cells, which was further reduced upon addition of the protonophore carbonyl cyanide 3-chlorophenylhydrazone (CCCP), suggesting that, in basal conditions, mitochondria from L75P-ApoA-I expressing cells still possess some residual function (Figure 1N).

These results suggest that the alteration in cellular proteostasis, presumably due to the prevalence of an amyloidogenic protein variant, affects the redox status of the cells while exacerbating mitochondrial stress and promoting cell death. Importantly, a similar alteration in redox homeostasis has also been described in a different experimental setup, i.e., cells treated with exogenously added fibrillogenic domain of ApoA-I. Indeed, Sakashita and colleagues demonstrated that when added to CHO and HEK293 cells, fibrils obtained from the N-terminal domain of ApoA-I carrying the G26R mutation were sufficient to induce ROS formation and mitochondrial dysfunction [33,34].

### 2.2. L75P-ApoA-I Induces an Impairment of the Autophagic Flux

**Figure 2:**
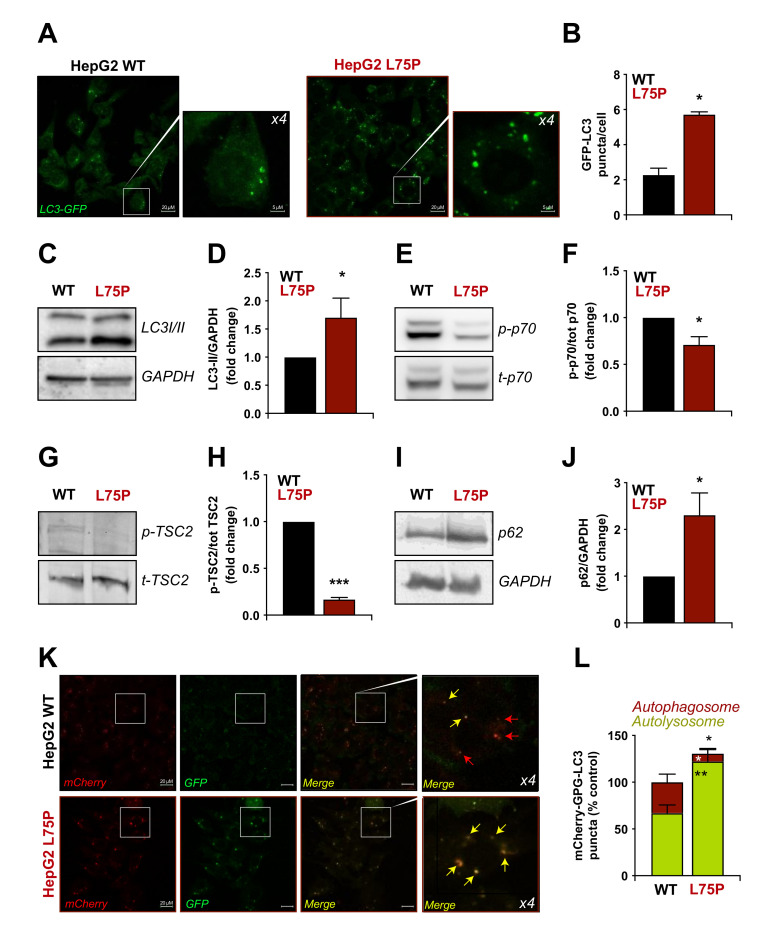
Measurement of autophagy in L75P-ApoA-I expressing cells. (**A**,**B**). Wild type (WT) and L75P-ApoA-I (L75P) HepG2 cells were transiently transfected with GFP-LC3, maintained in control conditions and the number of cytoplasmic GFP-LC3 dots per cell was quantified. (**C**–**J**). WT and L75P HepG2 cells were maintained in control conditions. Thereafter, LC3 lipidation (**C**), p70 (**E**) and TSC2 (**G**) phosphorylation as well as p62 levels (**I**,**J**) were quantified by immunoblot. GAPDH levels (**D**–**I**), total p70 (t-p70) (**E**) or total TSC2 (t-TSC2) (**G**) levels were monitored to ensure equal loading of lanes, and densitometry was employed to quantify the abundance of lipidated LC3 (LC3-II) (**D**), p70 (**F**) and TSC2 (**H**) phosphorylation and p62 (**J**). (**K**,**L**). WT and L75P HepG2 cells were transfected with the empty vector or pDest-mCherry-GFP-LC3B plasmid. After 24 h, the number of yellow vesicles (autophagosomes) and red vesicles (autolysosomes) was quantified. Images are from one experiment and representative of three independent experiments made in triplicates. If not previously specified, the panels in this figure are shown as means ± SD of at least three independent experiments. Asterisks symbols indicate significant changes with respect to WT cells (* *p* < 0.05, ** *p* < 0.01, *** *p* < 0.001).

Since autophagy is frequently altered in neurodegenerative disorders [35] and in (light chain) AL amyloidosis [36,37], we decided to monitor autophagic flux in our cell model system. To analyse basal autophagy, cells transfected with a green fluorescent protein (GFP)-MAP-LC3B (LC3) fusion construct were grown in complete medium for 48 h and analysed by fluorescent microscopy to detect GFP-LC3 puncta associated with autophagosomes or autolysosomes. Alternatively, the electrophoretic mobility of endogenous LC3 was determined in cell lysates analysed by immunoblotting. Upon induction of autophagy, the soluble cytoplasmic form of LC3 (LC3-I) undergoes an ATG4-mediated proteolytic cleavage, followed by conjugation to a phosphatidylethanolamine moiety on the autophagosomal membrane (LC3-II) *via* a ubiquitin-like conjugation system [38]. LC3 lipidation results in an increase in the number of GFP puncta (LC3 accumulating into membrane structures involved in autophagy) (Figure 2A,B) and an increase in electrophoretic mobility that allows monitoring the autophagic process (Figure 2C,D) [39].

Fluorescence microscopy (Figure 2A) revealed a 2.6-fold increase in the number of autophagic structures (detected as GFP-LC3 puncta/cell) (Figure 2B) in L75P-ApoA-I expressing cells transiently transfected with GFP-LC3 compared to control cells. This result was confirmed by immunoblotting analyses. Cells expressing the amyloidogenic variant had a significant increase in LC3-II/GAPDH ratio with respect to control cells (Figure 2C,D). Next, we investigated whether L75P-ApoA-I mutation induced autophagy is influenced by the mammalian target of rapamycin (mTOR) pathway, the main inhibitory regulator of the autophagy process (Figure 2E–H) [40]. The expression of L75P-ApoA-I variant inhibited the catalytic activity of mTOR in HepG2 cells, as assessed by the phosphorylation state of the mTOR substrate ribosomal protein S6 kinase, 70 kDa, polypeptide 1 (RPS6KB1, best known as p70S6K) (Figure 2E,F), as well as of the mTOR tonic inhibitor tuberous sclerosis 2 (TSC2) (Figure 2G,H) [40,41]. To determine whether L75P expression enhanced or hampered autophagic flux, we analysed the cellular levels of SQSTM1/p62 (Figure 2I), a LC3 interacting protein, which undergoes lysosomal degradation upon settings of autophagy induction [42]. Intriguingly, we found a 2.5-fold increase in p62 levels in cells expressing the amyloidogenic variant, compared to control transfected cells (Figure 2J), suggesting that autophagic flux is hindered in these experimental conditions. 

To further validate the result of the disruption of autophagic flux in cells with the L75P mutation, we took advantage of the mCherry-EGFP-LC3B construct that, based on the differential sensitivity to lysosomal pH of EGFP and mCherry (Figure S1A), allows us to faithfully quantify autophagic flux [43]. Upon mCherry-EGFP-LC3B transfection (Figure 2K), and as previously observed in Figure 2A–D, we observed an increase in the total number of yellow dots (red and green signal merged) in cells expressing the amyloidogenic variant compared to control cells (Figure 2L). Taken together, these results suggest that the L75P variant blocks the autophagic flux in HepG2 cells. Indeed, most of the puncta observed in the mutated condition were yellow (Figure 2L and Figure S1C,D), corroborating that lysosomal content is degraded in an ineffective fashion.

Interestingly, this impaired autophagic flux did not specifically depend on this specific amyloidogenic ApoA-I variant, since LC3II and p62 levels were also significantly increased in HepG2 cells expressing the L174S variant, responsible for preferential deposition of amyloid deposits in heart and skin (Figure S2A,B).

**Figure 3:**
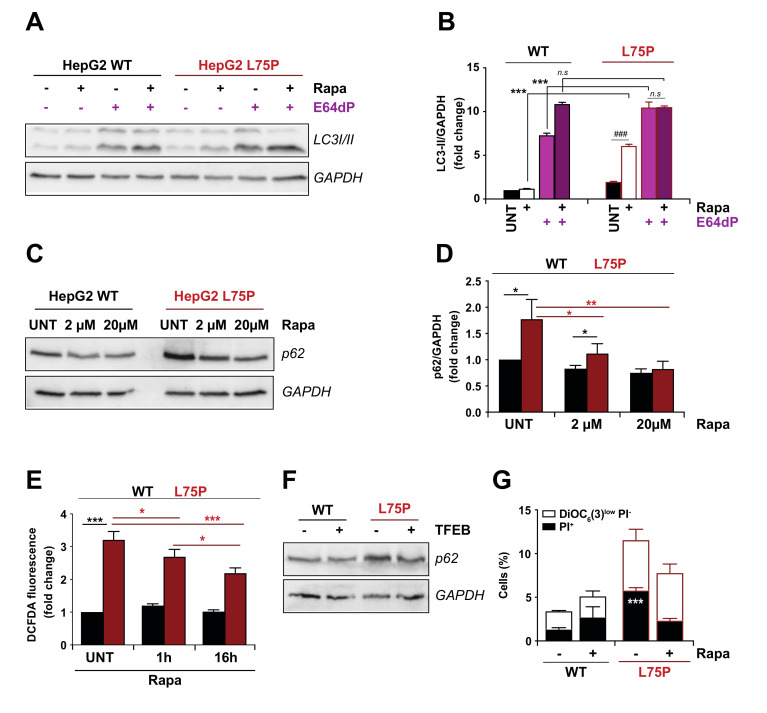
Effect of pharmacological induction of autophagy in L75P-ApoA-I expressing cells. (**A**–**D**). Autophagic flux evaluation. Wild type (WT) and L75P-ApoA-I (L75P) HepG2 cells were maintained in control conditions (UNT), treated with rapamycin (Rapa, 20 μM), alone or in combination with E-64d and pepstatin-A (E64dP) (5 μg/mL each) (**A**). Thereafter 24 h, LC3 lipidation was assessed (**B**). In other experiments, the cells were treated with increasing Rapa concentrations (2 or 20 μM) for 6 h and p62 levels were quantified (**C**). Densitometry in was employed to quantify the abundance of p62 (normalized to GAPDH levels) (**D**). (**E**) Intracellular oxidative stress was determinate as presence of cytosolic reactive oxygen species (ROS) and assessed by dichlorodihydrofluorescein (DCFDA) fluorescence intensity in WT and L75P HepG2 cells treated with Rapamycin (Rapa; 20 μM) for 1 or 16 h. (**F**) WT and L75P HepG2 cells were transfected with the empty vector or GFP-TFEB plasmid. 24 h after, the p62 levels were quantified. GAPDH levels were monitored to ensure equal loading of lanes. (**G**) Cytometric quantification of cell death after rapamycin treatment. WT and L75P HepG2 cells were treated, collected, and double stained with PI and DiOC_6_(3) for the detection of dying (DiOC_6_(3)^low^-PI^−^) and dead (PI^+^) cells. The immunoblot bands are from one experiment representative of three independent experiments. If not previously specified, the panels in this figure are shown as means ± SD of at least three independent experiments. Asterisks symbols indicate significant changes with respect to WT cells (* *p* < 0.05, ** *p* < 0.01, *** *p* < 0.001). Hash symbols indicate significant changes respect UNT condition (### *p* < 0.001).

In order to further unravel the issue whether autophagic flux was affected by L75P amyloidogenic variant, we incubated cells in the presence or absence of rapamycin (autophagy inductor acting as inhibitor of mTOR signalling and a potent enhancer of both autophagosome formation and clearance [44]) and presence or absence of the lysosomal protease inhibitors E64d and pepstatin A (E64d/PepA). We found that LC3II lipidation is increased in L75P expressing cells after rapamycin or lysosomal protease inhibitors treatments. However, in the presence of rapamycin and lysosomal proteases inhibitors together, the increased LC3 lipidation observed at basal levels in the mutated form disappeared (Figure 3A,B). This result confirms that autophagic flux is not totally blocked at basal levels (autophagic flux can be increased by rapamycin), but there is some alteration in autophagic flux, since LC3 lipidation cannot be increased when both treatments were combined.

We also used rapamycin to evaluate whether a pharmacological induction of autophagy in cells expressing the amyloidogenic protein would restore the autophagic flux in our system and if this could alleviate the vulnerability of cells to stress (Figure 1). In L75P-ApoA-I expressing cells, 24 h treatment with two different doses of rapamycin resulted into a decrease in p62 levels compared to L75P-ApoA-I untreated cells, but failed to restore p62 levels to baseline, indicating that rapamycin is able to partially reactivate protein degradation (Figure 3C,D). Moreover, ROS production induced by L75P amyloidogenic variant was partially abolished, in a time-dependent manner, when L75P-ApoA-I HepG2 cells were cultured in the presence of rapamycin (Figure 3E), suggesting that the hindered autophagy flux at basal levels is at least in part responsible for the oxidative stress observed in these conditions. This partial rescuing effect may be tied to the saturation of the lysosomal capacity to dispose of protein aggregates, a condition that is often associated with neurodegenerative disorders [45,46]. To evaluate this possibility, we transfected L75P-ApoA-I HepG2 cells and their wild type counterpart with the master transcriptional regulator of lysosomal biogenesis TFEB, which was previously shown to heighten lysosomal numbers and induce autophagy in basal or pathological conditions [47,48,49]. Importantly, we found that the enforced expression of TFEB in L75P-expressing cells restored p62 levels to those of wild type cells, suggesting that this manoeuvre normalizes autophagic flux (Figure 3F and Figure S3A,B). Finally, the high sensitivity observed in the L75P-expressing cells at baseline (Figure 1A–C) was reversed treatment with rapamycin (Figure 3G).

## 3. Discussion

Taken together, these data indicate that the expression of the L75P-ApoA-I variant blocks autophagic flux, which is at least in part responsible for a compromised cellular proteostasis and oxidative stress, two factors that ultimately lead to cell death in mutated cells. Since cellular proteostasis is primarily affected in amyloidogenic diseases, we speculate that specific alterations in the autophagic pathway might play a central role in the pathogenesis of ApoA-I related amyloidosis. Accordingly, it has been reported that altered expression level of ApoA-I affects autophagy in hepatic steatosis. Indeed, the modulation of the abundance of ApoA-I in HepG2 cells and in mice promotes hepatic autophagy through the AMPK-mTOR pathway, and, consequently, prevents cell death and reduces steatohepatitis [24,25].

Interestingly, lysosomal dysfunction and autophagy impairment were also found in HEK293 cells treated with the fibrillogenic domain of ApoA-I carrying the G26R mutation [31], indicating that these phenomena can be induced by both the fibrils precursors, i.e., the full-length mutated form of ApoA-I, and fibrils themselves. To date, there is no experimental proof supporting the hypothesis that L75P-ApoA-I would form intracellular oligomers in hepatic cells. However, there are several pieces of evidence that could link the observed lysosomal dysfunction to protein variant oligomers formation. Indeed, it should be noticed that: (i) HepG2 cells expressing the L75P variant retain a higher amount of ApoA-I, compared to cells expressing the WT protein [19]; (ii) the recombinant L75P variant is more susceptible to misfolding upon environmental changes compared to the native protein [11]; and (iii) an acidic environment is known to enhance the aggregation propensity of ApoA-I [50]. Based on these observations, it is tempting to speculate that since the L75P variant has a misfolding-prone structure, it is readily redirected to the lysosomes for degradation. However, the acidic environment of lysosomes may foster L75P aggregation, rendering the protein resistant to the proteolytic cleavage. This would explain the accumulation of protein inside the cells. This speculation is strengthened by the findings from Jayaraman and colleagues, who reported that serum amyloid A is able to form stable proteolysis-resistant soluble oligomers at lysosomal pH. These oligomers accumulate in the lysosomes and, eventually, damage cellular membranes, thus releasing intracellular amyloids into the extracellular space [51]. Moreover, the preferential accumulation of ApoA-I variants in specific tissues are not yet fully uncovered. It is possible that the specific interaction of the circulating ApoA-I variants with specific components of cell membranes or of the extracellular matrix has an effect of the structure and stability of the variants and drives the preferential tissue accumulation. Indeed, it has been reported that specific cell milieus can affect the conformation and aggregation propensity of L75P and L174S ApoA-I amyloidogenic variants in a different, specific way [52].

Another aspect to consider is that while most of the ApoA-I is secreted in its lipid-free or lipid-poor form, about 20% of ApoA-I newly secreted from HepG2 cells is already lipidated, and it is in the form of nascent HDL particles [53]. However, the amount of newly secreted lipidated ApoA-I might be different in the case of amyloidogenic variants. Indeed, our previous studies indicated that ApoA-I variants, and L75P in particular, had a much lower affinity for the lipids and that the equilibrium between lipid-bound and lipid-free/poor forms is shifted towards the latter [54]. We also demonstrated that the lipidated L75P variant possess a more compact structure and is more stable than their lipid-free counterpart [14]. This allows us to speculate that it is the intracellular lipid-free form of L75P that causes the autophagy impairment here described.

Of note, strategies aiming to reactivate the autophagic flux (as treatment with the mTOR inhibitor rapamycin) and increase the number and function of lysosomes (as in the case of TFEB overexpression) may represent a successful translational approach to mitigate the amyloidogenic phenotype.

## 4. Materials and Methods

### 4.1. Cell Lines, Culture Conditions and Treatments

Human hepatocellular carcinoma (HepG2) cell lines were purchased from ATCC. HepG2 cells stably expressing the amyloidogenic variant L75P-ApoA-I were obtained as previously described [19]. Cells were maintained at 37 °C in 5% CO_2_ in Dulbecco’s Modification of Eagle’s Medium (DMEM) supplemented with 10% foetal bovine serum (FBS), 1% penicillin/streptomycin and L75P-ApoA-I and L174S-HepG2 cells were grown in the presence of 0.8 mg/mL G418. Cells were plated on six-well plates at 3 × 10^5^ cells/well. After 24 h, the medium was replaced with fresh complete medium, and the cells were treated with rapamycin (2 or 20 μM for 6h; 20 μM for 1 h or 16 h) in the presence or absence of lysosomal inhibitors E64d and pepstatin-A (Pep-A) (5 μg/mL each) for the indicated time at 37 °C. At the end of incubation, cells were detached with trypsin, lysed and analysed by Western blotting. In case of serum starvation, cells were incubated in DMEM without FBS for the indicated time. Unless otherwise specified, chemicals were purchased from Sigma-Aldrich (St. Louis, MO, USA), culture media and supplements for cell culture from Gibco-Life Technologies TM (Carlsbad, CA, USA) and plasticware from Corning Inc. (Corning, NY, USA).

### 4.2. Plasmids Transfection

Cells (4 × 10^4^ cells/cm^2^) were seeded on glass coverslips in 24-well plates. After 24 h, transient transfections were performed with Lipofectamine 2000 reagent (Invitrogen, Carlsbad, CA, USA). Cells were transfected with empty vector, alone or together with GFP-LC3 [55], pDest-mCherry-EGFP-LC3B (kindly provided by Prof. Terje Johansen, Molecular Cancer Research group, Institute of Medical Biology, University of Tromsø, Norway) or pEGFP-N1-TFEB (gift from Shawn Ferguson, Addgene plasmid # 38119, Cambridge, MA, USA) plasmids. After 5 h, the transfection medium was replaced with fresh culture medium, and cells were incubated until the following day for preventing cellular stress and optimizing transfection. All images were taken using a Zeiss LSM 700 (Oberkochen, Germany) confocal microscope under identical exposure conditions or an imaging flow cytometer ImageStream^®^ (Amnis^®^ Flow Cytometry, Luminex, Seattle, WA, USA). Images were analysed with Image J software. 

### 4.3. Western Blot

At the end of the experiment, cells were detached by trypsinization and lysed in lysis buffer (100 mM Tris HCl pH 7.4, 300 mM NaCl, 0.5% NP40, protease inhibitor cocktail, phosphatase inhibitors). Upon 30 min incubation on ice, lysates were centrifuged at 14,000× *g* for 30 min at 4 °C and supernatants were collected. Following the determination of protein content by the Bradford assay, 50 μg of proteins were separated by SDS-PAGE and analysed by Western blotting using specific antibodies. Antibodies from Cell Signalling (Danvers, MA, USA) were used at 1:1000 dilution: anti-phospho Akt (#4060S; lot: 16), anti-phospho p38 (#4111P; lot: 10), anti-phospho SAPK/JNK (#4668P; lot: 11), anti-caspase-3 (#9664P; lot: 20), anti-phospho TSC2 (#3617; lot: 2), anti-LC3 (#2775S; lot: 5), anti-phospho p70 (#9205; lot:16), anti-p70 (#9202; lot: 14), anti-TSC2 (#3612; lot: 3). The anti-p62 was from Novus Biologicals, Littleton, CO, USA; 1:5000 dilution; #H00008878-M01; lot: FC071-2C11); anti-GAPDH (Thermo Scientific, USA; 1:10000 dilution, #PA1-988; lot: OH188198); anti β-Actin (Sigma-Aldrich; 1:1000 dilution, #A4700; lot: 036H4857). Detection was performed according to the manufacturer’s instruction, using a ChemiDoc (Biorad, Hercules, CA, USA). 

### 4.4. DCFDA Assay

For the determination of the cytosolic ROS levels, cells were plated at 1 × 10^6^ cells/well in a 10 cm plate at 37 °C. After 24 h, cells were incubated with the cell permeable, redox-sensitive fluorophore 2′,7′-dichlorodihydrofluorescein diacetate (H_2_-DCFDA, Sigma-Aldrich) following the procedure previously described [56]. The DCF fluorescence intensity was measured using a PerkinElmer LS50 spectrofluorometer (emission wavelength of 525 nm and an excitation wavelength of 488 nm, scanning speed of 300 nm/min, with 5 and 5 slit widths for excitation and emission, respectively). ROS production was related to DCF fluorescence intensity.

### 4.5. TMRE Assay

To analyse mitochondrial membrane potential (Δψm), the protocol previously described [57] was followed. Briefly, cells were plated at a density of 2 × 10^4^ cells/well and after 24 h treated as described above. At the end of treatment, cells were incubated with 200 nM of cationic lipophilic dye tetramethylrhodamine ethyl ester (TMRE) for 20 min at 37 °C. Then, cells were gently washed with PBS with 0.2% BSA for three times and the fluorescence was measured in a microplate reader with peak Ex/Em = 549/575 nm. Each value is the mean of three independent experiments, each with three determinations. In other experiments, cells were stained with 150 nM tetramethylrhodamine methyl ester (TMRM, from Molecular Probes-Life Technologies) and 5 µg/mL 4′,6-diaminidino-2-phenylindole (DAPI, from Molecular Probes-Life Technologies) for 30 min at 37 °C. Cytofluorometric determinations were carried out on a MACSQuant cytometer (Miltenyi Biotec, Bergisch Gladbach, Germany). Cells were incubated with pre-warmed medium containing 500 nM MitoTracker (Molecular Probes-Life Technologies) and 5 µg/mL DAPI for 1 h at 37 °C. Thereafter, cells were rinsed twice with pre-warmed medium, and processed for cytometry.

### 4.6. Cytofluorometric Assessment of Apoptosis

For apoptosis determinations, after the indicated treatments, cells were collected and co-stained for 30 min at 37 °C, with 1 mg/mL of propidium iodide (PI) (Sigma-Aldrich), which identifies cells with ruptured plasma membranes, and 20 nM DiOC_6_(3) (Molecular Probes-Life Technologies) for the cytofluorimetric detection of dying (DiOC_6_(3)^low^ PI^−^) and dead (PI^+^) cells, as previously described [58]. Cytofluorometric acquisitions were performed on an Attune Ntx Flow Cytometer (ThermoFisher Scientific, Waltham, MA, USA).

### 4.7. Statistical Analyses

Unless otherwise mentioned, data are reported as mean ± SD/SEM of triplicate determinations and experiments were repeated at least twice yielding similar results. Data were analysed using Prism 5 (GraphPad Software, Inc., La Jolla, CA, USA) or Excel (Microsoft Co., Redmond, WA, USA), and statistical significance was assessed by means of two-tailed Student’s t-test or ANOVA tests, as appropriate.

## Data Availability

The data presented in this study are available on request from the corresponding author.

## Supplementary Materials

The following supporting information can be downloaded at: https://www.mdpi.com/article/10.3390/ijms23073498/s1.

## Abbreviations

Akt	protein kinase B
AL	light chain
ApoA-I	apolipoprotein A1
Atg	autophagy-related
BSA	bovine serum albumin
DMEM	Dulbecco’s Modification of Eagle’s Medium
FBS	foetal bovine serum
G418	geneticin
GAPDH	glyceraldehyde 3-phosphate dehydrogenase
GFP	green fluorescent protein
H_2_-DCFDA	2′,7′-dichlorodihydrofluorescein diacetate
HepG2	human hepatocellular carcinoma
LC3	microtubule associated protein 1 light chain 3 (MAP1LC3)
Leu	leucine
MAPK	mitogen-activated protein kinase
MTOR	mammalian target of Rapamycin
p70	ribosomal protein S6 kinase beta 1 (RPS6KB1, p70S6K)
PE	phosphatidylethanolamine
Pep-A	pepstatin-A
Pro	proline
ROS	reactive oxygen species
SAPK/JNK	stress-activated protein kinase/Jun-amino-terminal kinase
SD	standard deviation
SEM	standard error of the mean
SQSTM1/p62	sequestosome 1
TFEB	transcription factor EB
TMRE	tetramethylrhodamine ethyl ester
TSC2	tuberous sclerosis 2
UV	ultraviolet
WT	wild-type
Δψm	mitochondrial membrane potential
