# Supplementary material for: Autophagy Alteration in ApoA-I Related Systemic Amyloidosis
Source: Int J Mol Sci. 2022 Mar 23;23(7):3498. doi: 10.3390/ijms23073498 (PMC8998969; doi:10.3390/ijms23073498)
Supplement: Supplementary file 1 [file ijms-23-03498-s001.zip › ijms-1610290-supplementary (1).pdf]

Supplementary Materials

Autophagy alteration in ApoA-I related systemic amyloidosis

Rita Del Giudice [1,†], Paola Imbimbo [1,†], Federico Pietrocola [2], Isabelle Martins [3,4], Fatima Domenica Elisa De Palma [3,5,6], José Manuel Bravo-San Pedro [7], Guido Kroemer[3,4,8,9], Maria Chiara Maiuri [3,4,10] and Daria Maria Monti [1,11,*]

1 Department of Chemical Sciences, University of Napoli Federico II, Complesso Universitario Monte Sant'Angelo, Napoli, Italy; rita.del-giudice@mau.se (R.D.G.); paola.imbimbo@unina.it (P.I.)

2 Department of Biosciences and Nutrition, Karolinska Institute, Huddinge, Sweden; federico.pietrocola@gmail.com

3 Centre de Recherche des Cordeliers, INSERM U1138, Université Paris Cité, Sorbonne Université, Paris, France; isabelle.martins@gustaveroussy.fr (I.M.); depalma@ceinge.unina.it (F.D.E.D.P.); kroemer@orange.fr (G.K.); chiara.maiuri@upmc.fr (M.C.M.)

4 Metabolomics and Cell Biology Plaforms, Gustave Roussy Cancer Campus, Villejuif, France

5 CEINGE-Biotecnologie Avanzate s.c.a.r.l., Napoli, Italy

6 Department of Molecular Medicine and Medical Biotechnologies, University of Napoli Federico II, Napoli, Italy

7 Universidad Complutense de Madrid, Departamento de Fisiologia, Madrid, Spain; chemabsp@gmail.com

8 Institut Universitaire de France, Paris, France;

9 Pôle de Biologie, Hôpital Européen Georges Pompidou, Ap-hp, Paris, France ;

10 Pharmacy Department, University of Napoli Federico II, Napoli, Italy

11 Istituto Nazionale di Biostrutture e Biosistemi (INBB), Rome, Italy

* Correspondence: mdmonti@unina.it

† These authors equally contributed

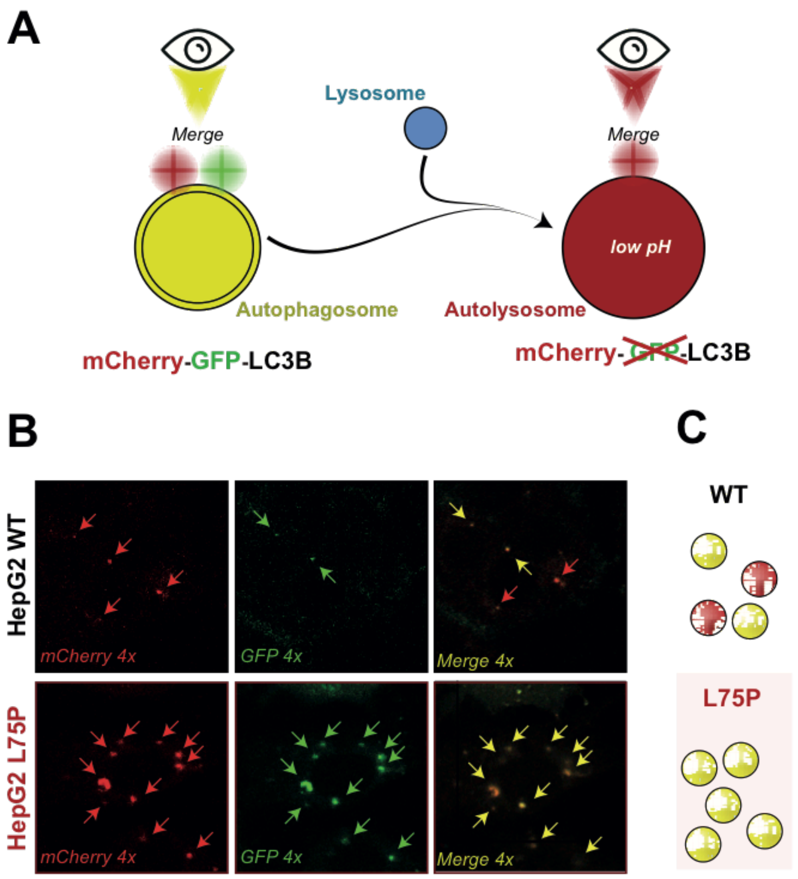

Figure S1. The mCherry-GFP-LC3 tandem fluorescent probe (**A**). Schematic representation of mCherry-GFP-LC3 tandem fluorescent probe emit yellow fluorescence (green-red merged) in non-acidic structures (phagophores or autophagosomes) and emit in red in the autolysosomes due to quenching of GFP in low pH conditions. (**B, C**). 4x magnification of the pictures shown in the Figure 2K (**B**) and summary (**C**) of the results showed in **Figure 2L**. The red arrows show the fluorescent signal of mCherry, the green arrows show the fluorescent signal of GFP and the yellow arrows show both signals merged. In merged image, the red arrows indicate autolysosomes (green signal has been lost in contact with acidic pH) and the yellow arrows indicate the presence of autophagosomes (green signal is intact).

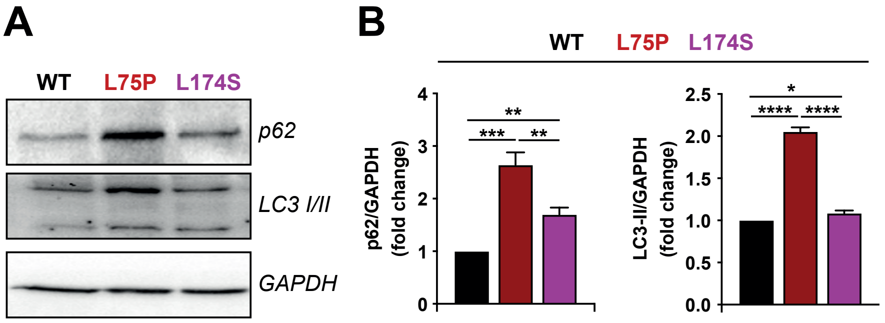

Figure S2. Measurement of autophagy in L174S-ApoA-I expressing cells. Wild type (WT), L75P-ApoA-I (L75P) and L174S-ApoaA-I (L174S) HepG2 cells were maintained in control conditions. Thereafter, the LC3 lipidation and p62 expression levels were evaluated by immunoblot (**A**). GAPDH levels were monitored to ensure equal loading of lanes, and densitometry was employed to quantify the abundance of lipidated LC3 (LC3-II) and p62 (both normalized to GAPDH levels) (**B**). One representative experiment out of three performed is showed. Data are means ± SD of at least three independent experiments; *$p < 0.05$, **$p < 0.01$, ***$p < 0.001$, ****$p < 0.001$.

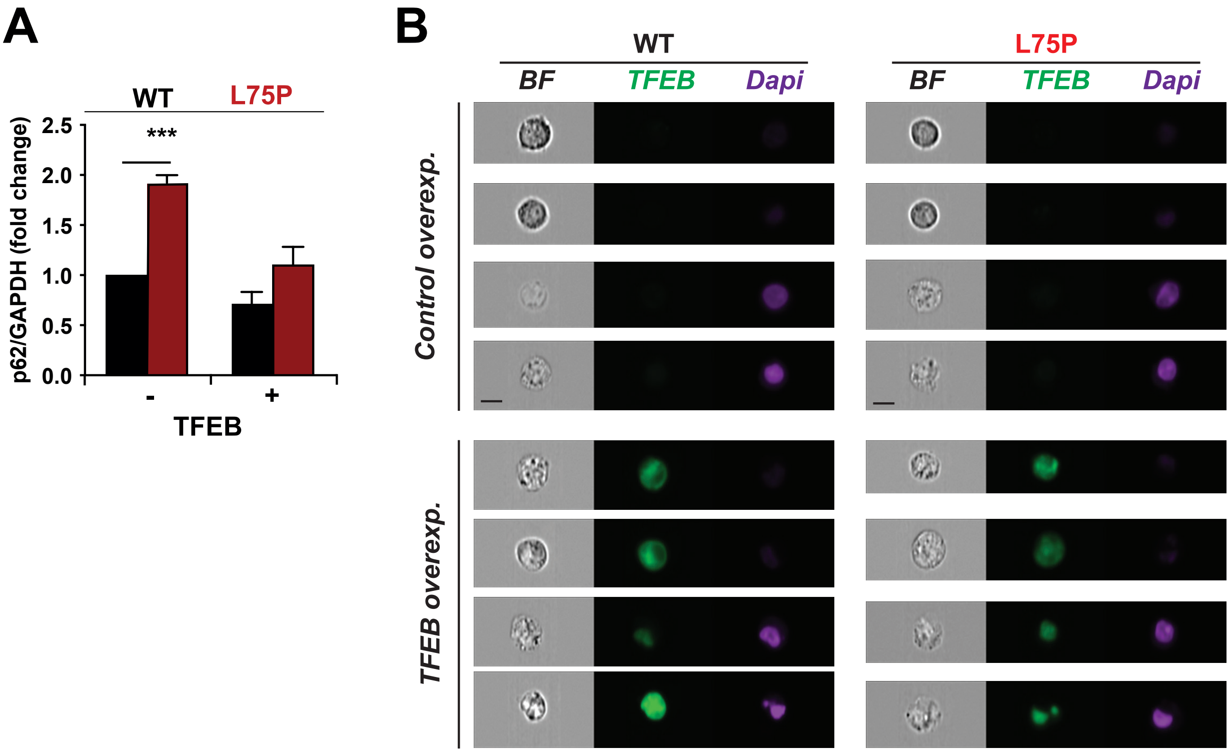

Figure S3. GFP-TFEB plasmid transfection. (**A**) Densitometry of immunoblot showed in **Figure 3F**, employed to quantify the abundance of p62 (normalized to GAPDH levels). Data are means ± SEM of at least three independent experiments; ***p < 0.001. (**B**) Representative images showing Wild type (WT) and L75P-ApoA-I (L75P) HepG2 cells transfected with the empty vector or GFP-TFEB plasmid.
